# Some Dangers and Mistakes to Be Avoided

**Published:** 1914-08-22

**Authors:** 


### A STATE OF WAR.

### THE COUNTRY AND THE CARE OF THE SICK AND WOUNDED.

### Some Dangers and Mistakes to be Avoided.

Everybody throughout the United Kingdom—that is, not only the representatives, but the individual members of every class of the community—show the most encouraging and earnest desire to be helpful in meeting the requirements which the nation as a whole must fulfil to prevent any failure to meet the demands for hospital, medical, and nursing care arising from the present state of war. Lord Rothschild, the Chairman of the Council of the British Red Cross Society, in a letter which appeared in the *Times* last Saturday, sounds a note of warning against avoidable dangers and mistakes: "We are threatened with the same confusion that so crippled Red Cross effort in the South African war, with the same evils of overlapping, of unco-ordinated and disunited work. Private houses are being turned into hospitals and convalescent homes without reference to any organising body and without regard to any rational scheme. Nurses are engaged who may never be required in the particular place allotted to them, while, worst of all, stores of surgical material are being hoarded up in scores of houses to such an extent that the market is seriously depleted. Ladies are starting independent base hospitals of their own, and are appealing for funds for their maintenance. Surgeons and nurses are struggling to reach the front without any organisation and without definite orders or definite plans. All these efforts are most kindly meant, but they are producing an amount of disorder and waste of *personnel* and equipment which is to be deplored. The British Red Cross Society has already a number of beds at its disposal which, while probably sufficient to meet the coming need, can be almost indefinitely expanded on efficient and economic lines. The organisation of the Society extends throughout the whole country, and beds can be provided in sufficient number as and when they are required."

#### The British Red Cross Society.

Lord Rothschild's declaration is timely, and it is encouraging to know that the British Red Cross Society should be so fully alive to the dangers arising from a want of co-ordination and to have his assurance that the British Red Cross Society is working in close harmony with the St. John Ambulance Association. The British Red Cross Society is keeping in close touch with the War Office and Admiralty, to whom alone they look for instructions as to where help is needed, and as to the nature of such help. All but the clerical work of administration of the Red Cross Society Lord Rothschild declares is carried out by trained volunteers, every department is in charge of competent experts, while offices and store-rooms are provided free of all cost by the generosity of the Duke of Devonshire. All this is most encouraging and satisfactory.

#### Ways and Means.

We gather from Lord Rothschild's letter that the British Red Cross Society is in need of much more money to carry out on business lines its mission of mercy, the organisation of which has been the deliberate work of years. The expenses for equipment, for *personnel*, and for supplies must needs be heavy, and although the public who subscribe to the British Red Cross Society funds have the assurance that every penny contributed will be employed in the direct and proper relief of the sick and wounded, it is certain that the Government ought to underwrite the whole expenses of this Society from the outset, so that it may be in a position to incur promptly every necessary expenditure, or delays, and worse, may be caused, to the detriment of the work and the danger of the sick and wounded. Seeing that the naval and military authorities in time of war delegate to the Red Cross Society the task of organising and co-ordinating voluntary aid, including the provision of voluntary and temporary hospitals throughout the country, such delegation ought certainly to rest upon the sound financial basis of a Government guarantee for all the necessary expenditure incurred. Such a guarantee would not in any way diminish, but would rather tend to increase, in our judgment, the free offerings of the public, providing adequate organisation for the raising of funds is set on foot in conjunction with central committees working in the chief city or the principal towns of each county throughout England and Wales. The organisation of voluntary help and of the supply of voluntary and temporary hospital and semi-convalescent accommodation should be closely identified with the raising of funds locally to defray the extra cost incurred. By such a plan the interest of every resident in every county might readily be secured to lend a hand, and the total result must prove exceedingly gratifying. We are glad to know that the city of Birmingham has taken action in this direction, and that both the local Press and the principal inhabitants are fully alive to the fact that such an organisation should be set on foot forthwith.

#### Much Accommodation may be Needed.

Members of the medical profession who are engaged on service have conveyed to us the view that the amount of accommodation required for the sick and wounded may be great, and they are impressed with the feeling that the nature and character of the severer injuries to be dealt with may be unusually large. These considerations make it of the first importance that the number of hospital beds —that is, of beds provided by recognised hospitals possessed of the fullest equipment and so capable of handling these cases—should be adequate. To make them adequate may prove a serious matter for the civil population of these islands, and it is of the first importance that the many questions arising out of such a provision, and the best way to meet the difficulties which can be foreseen, should be taken in hand at once and planned under a definite, well-thought-out scheme.

### What has been done.

Already practical steps have been taken in this important matter. We owe the existing organisation to the inspiration and ability of Sir Alfred Keogh, late Medical Director-General of the Army Medical Service. In 1907 he evolved a practical and extended plan which has steadily been developed. Under his direction steps were taken (*a*) for the establishment in selected buildings throughout the country of twenty-three general hospitals containing a minimum of 500 beds, (*b*) to set up and to make provision for an adequate medical and nursing staff locally for each such hospital. Many of the buildings selected had ample space surrounding them, so that in some cases it might be possible to provide in the adjacent grounds further hospital accommodation for from 1,000 to 1,500 extra patients. The selection of suitable buildings, mainly public buildings, was decided upon in substituton for the tent hospitals originally contemplated. The organisation of these hospitals has been carried out by the Royal Army Medical Corps, and also their equipment. For a list of these hospitals see The Hospital, August 15, 1914, page 553.

It reflects great credit on the department of the Medical Director-General of the Army, especially in view of the attempts which are being made to raise the price of bedsteads and other necessary articles of hospital equipment, that most things necessary to furnish and open each of these hospitals were purchased and stored in sections, so that it has been relatively easy to transfer each complete equipment from the stores to the Territorial hospital to which it was registered and assigned. Attached to each hospital there will be an adequate ambulance service, so that the sick and wounded may be readily conveyed to the hospitals and, as they convalesce, from them to the semi-convalescent and other health-restoring accommodation, which it is hoped to provide in connection with every base hospital.

Again, before the war was thought of the Medical Departments of the Navy and of the Army decided that it was essential to organise a reserve auxiliary through Queen Alexandra's Imperial Military Nursing Service. Returns were obtained after consultation with the managers and matrons of the principal voluntary hospitals throughout the country. A special department was formed at the War Office, and Miss Sidney Browne, R.R.C., was appointed matron-in-chief and placed in charge of it. This resulted in the establishment of a high standard of efficiency from the outset, and that efficiency has been maintained by Miss Becher, R.R.C., the present matron-in-chief. There are at present upwards of twenty military hospitals, including those in Great Britain and Ireland, with an organised body of sisters and staff nurses. In addition to these military hospitals there has been established comparatively recently in connection with the Territorial Nursing Service, which is now under the charge of Miss Sidney Browne, an organisation for hospital accommodation in the voluntary hospitals. We published a list of these Territorial hospitals on page 553 of The Hospital last week.

### Port Provision.

We are glad to be assured that the military and naval authorities have established a system which has secured that at every English and Scottish port to which a ship with wounded men may come there will be an adequate hospital ready for the reception of the wounded, be they sailors or soldiers. It is fair to conclude, therefore, that initially the military, naval, and Territorial hospital accommodation will be found reasonably adequate. We hope this may prove to be so, but we are strongly impressed with the belief that there is at present great need for a supplementary organisation to provide a chain of semi-convalescent hospitals without delay, so that they may be ready to help the base hospitals by relieving them of the wounded directly they are sufficiently recovered after operation to render their removal to the country desirable, where their complete recovery may be expedited by a month's rest in the open air. We deal later on with the class of accommodation and the best way of organising this chain of semi-convalescent institutions.

### Hospital Ships.

It may be well to emphasise the statement which has been published to the effect that the Union-Castle liners, *Carisbrooke Castle* and *Dunvegan Castle*, of upwards of 7,000 and 6,000 tons respectively, have been fitted up as hospital ships, and they are now stationed where the naval and military authorities consider they are likely to prove most helpful when occasion arises. Here we may point out that the presence at the moment of a number of yachts and smaller vessels for the accommodation of our wounded in home waters might possibly be an encumbrance rather than a help or benefit to the Navy. The responsibility and decision for the use or rejection of such smaller vessels in the present war is one which the naval authorities alone can undertake.

### Essentials to Complete Success.

The first essential to the complete success of the temporary provision for the relief of the sick and wounded throughout the country is that the whole organisation on which it must depend for success should be promptly thought out and completed. This organisation must include the setting up of definite standards of efficiency and equipment. For instance, even the goodwill and anxiety of everybody to help in the work is not free from danger. It unavoidably tends at the outset to cause an immense amount of trouble, to create disappointment, and, unless definite decisions are taken promptly upon material matters, the perfecting of this service so as to make it worthy of the nation, and equal to the standard of accommodation which ought to characterise the equipment and working of every auxiliary home hospital and semi-convalescent hospital, may become impossible of attainment. In the ambition to do something many of the managers of the smaller institutions hurry to send in offers of so many beds in a relatively small institution, no matter where it may be situated. In the case of other hospitals where the will is present to render good service, and more businesslike and prudent counsels prevail, the managers are anxious to know if any, and what, grants in aid will be made by the Red Cross Society.

These two instances out of a hundred we might quote will suffice to enable our meaning to be clearly grasped. If a standard of requirement and efficiency were to be formulated, which standard must be attained as a minimum qualification to all who wish to offer accommodation, and the technical conditions on which acceptance will be based were to be settled, their publication would remove all doubts, and would lessen immensely the work entailed by the sending in of innumerable offers of assistance which on the face of them cannot be entertained. The same remark applies to the whole organisation of an adequate system of temporary auxiliary home hospitals and semi-convalescent accommodation. Details should be laid down as to locality, the amount of vacant land contiguous to the buildings offered, the quality and nature of the sanitary and hygienic conveniences; these and a number of other practical details with full particulars must be forthcoming, before it will be wise, if it is even possible, to express an opinion as to whether this or that building or accommodation can be accepted for the purposes of the British Red Cross Society's work.

We have seen types of the kind of equipment which has been hurriedly got together for so-called auxiliary hospitals, which presumably are intended by their well-intentioned promoters to form a portion of the organisation for which the British Red Cross Society will assume responsibility. We have noticed that the beds and bedding attain no standard, that they have been got together from a number of people who have been asked to contribute beds or bedding or equipment of various kinds in accordance with a long list which has been circulated, very properly, by local committees of the Red Cross Society in country districts, but which seems to us to be altogether unsuited to the work of a British Red Cross Committee situated in and in charge of an area which represents a large and wealthy metropolitan borough. Yet the latter has circularised the residents in a large district like Kensington, to name one instance known to us, and the receipt of its circular has caused amazement to many recipients, who have no knowledge as to the kind of articles which would be most suitable for a large hospital's requirements. Some of these recipients have pointed out to us that they would be very glad indeed to send a cheque to defray the cost of so many beds, or of certain sections of equipment, provided they were supplied with information as to the cost entailed.

We would venture to suggest, therefore, to the Committee of the British Red Cross Society, that they should have prepared, without delay, accurate lists setting forth the cost of each article, and that then an appeal should be made to each of the wealthier households in each district to contribute a definite sum. As matters stand at present, in fulfilment of the general wish to help without accurate knowledge or adequate apprehension, we imagine that some committees may have been flooded with a mass of articles and materials which could not properly be used in any auxiliary hospital, if it is to attain the standard of equipment which every establishment for which the British Red Cross Society may be responsible must attain if it is to be worthy of the occasion, and able efficiently to provide every comfort and hygienic appliance which our wounded soldiers and sailors have a right to find ready for them when the need arises.

We have only indicated in outline what we mean by expressing a hope that definite standards and necessary details and instructions will be prepared for the guidance of all who wish to give or to help forward the speedy and adequate equipment of every auxiliary home hospital and semi-convalescent institution which the British Red Cross Society have now the heavy responsibility of providing. No one can be more conscious than we are of the serious responsibility which rests upon the British Red Cross Society at the present time, for on their shoulders will be laid the faults of any miscarriage which may occur, even where the circumstances may have been unforeseen. This consideration makes it the more imperative for everyone who can render practical assistance to this Society to place it without delay at their disposal. Our point is that brains, knowledge, and careful forethought are forces which, when applied in their full strength, initially, can effectually prevent failure in any branch for which the British Red Cross Society may be directly responsible.

### British Red Cross Society.

We feel it may be helpful to all who are interested in some of the many fields of work now under the direction of the Red Cross Society that we should publish the following list, giving the heads of the different departments, and those who have been appointed to take charge of and direct them:—

*Personnel.*—Sir Frederick Treves, G.C.V.O.

*Convalescent Homes* (Joint Committee with the Soldiers' and Sailors' Help Society).—Princess Christian, Sir Rowland Bailey, Lady Northcote,

Major-General Lord Cheylesmore, Major Tudor Craig, Lord Grenfell, Georgiana Countess of Dudley.

*Medical Comforts and General Gifts of Stores.*—Hon. A. Stanley, Lady Ampthill, Lady Wolverton.

*Hospitals and Red Cross Assistance Abroad* (Stores, Equipment and Staff).—Sir A. Bowlby, Lady Perrott.

*Arrangements Necessary for the Transport of Red Cross Assistance Overseas to the British Army.*—Sir W. Garstin, G.C.M.G., Captain W. J. Lyons, Lord Onslow.

*Arrangements Necessary for the Transport of Red Cross Assistance to our Allies.*—Major the Hon. R. White, 16 Stratton Street (7775 Gerrard).

*Auxiliary Home Hospitals.*—George Henry Makins, Esq., C.B., Dr. Edward Stewart.

*Hospitals and Ambulance Ships.*—Sir Frederick Treves, G.C.V.O., Dr. Edward Stewart.

*Motor and Ambulance Transports.*—Sir Frederick Treves, G.C.V.O.

*Finance.*—The Hon. N. Charles Rothschild, E. A. Ridsdale, Esq.

*The Distribution of Medical and General Comforts to Home Hospitals.*—Horace Folker, Esq., H. W. Laurie, Esq., 27 Harrington Gardens, S.W.

*Office.*—Sir Walter Lawrence, Bart.

The Central Offices of this Society are at Devonshire House, Piccadilly, London, W.

